# Dr. Lemuel Bolton Bangs

**Published:** 1914-11

**Authors:** 


Dr. Lemuel Bolton Bangs, P. & S., 1872, died at his home in New York, October 4, aged 72. He was well known as a surgeon and writer.

